# Genetic polymorphism (rs6776158) in CaSR gene is associated with risk of nephrolithiasis in Chinese population: Erratum

**DOI:** 10.1097/MD.0000000000013755

**Published:** 2018-12-14

In the article, “Genetic polymorphism (rs6776158) in CaSR gene is associated with risk of nephrolithiasis in Chinese population”,^[1]^ which appears in Volume 97, Issue 45 of *Medicine*, affiliation a should be “Department of Urology, Yangzhou No.1 People's Hospital, Affiliated Hospital of Yangzhou University, Yangzhou, China.”
